# Effect of experimental conditions on size control of Au nanoparticles synthesized by atmospheric microplasma electrochemistry

**DOI:** 10.1186/1556-276X-9-572

**Published:** 2014-10-14

**Authors:** Xunzhi Huang, Yongsheng Li, Xiaoxia Zhong

**Affiliations:** 1 State Key Laboratory of Advanced Optical Communication Systems and Networks, Key Laboratory for Laser Plasmas (Ministry of Education), Department of Physics and Astronomy, Shanghai Jiao Tong University, Shanghai 200240, China

**Keywords:** Microplasma, Electrochemistry, Nanoparticle

## Abstract

Atmospheric microplasma electrochemistry was utilized to synthesize Au nanoparticles (NPs). The synthesized Au NPs were investigated as a function of reduction current, solution temperature, and stirring (or not) by using ultraviolet-visible (UV-Vis) absorbance and transmission electron microscopy (TEM). It was illustrated that high current promoted the growth of Au NPs with small size, and more Au NPs with large size were synthesized as a rise of temperature. The Au NPs often with small size were synthesized as a result of stirring. The production rate, the electrostatic repulsion, and the residence time of the Au NPs at the interfacial region play an important role in the growth of Au NPs. The results shed light upon the roadmap to control the size and particle size distribution (PSD) of Au NPs synthesized by atmospheric microplasma electrochemistry.

## Background

The preparation of noble metal nanoparticles (NPs) can be traced back to ancient times in mid-nineteenth century [1]. The preoccupation of scientists with NPs preparation remained unabated, primarily by their fascinating properties and potential applications in catalysis [2-6], biology [6-10], optics [11,12], electronics [13,14], solar cells [15], sensing [16-18], and medicine [6,19]. Among the various methods operated for the preparation of noble metal NPs [20-30], recently reported atmospheric microplasma electrochemistry (plasma-liquid electrochemistry or gas-liquid interface discharges) is the most reliable and attractive one, on account of its advantage in metal NPs production such as fast and environment-friendly (any toxic-reducing agents or stabilizers such as NaBH and TBAB [23,29] are not involved).

In this paper, we studied the particle size and the particle size distribution (PSD) of the Au NPs synthesized by atmospheric microplasma electrochemistry under different experimental conditions. The variation in the particle size and the PSD of Au NPs with current, temperature, and stirring (or not) were demonstrated via transmission electron microscopy (TEM) and ultraviolet-visible (UV-Vis) spectroscopy and were explained in terms of the production rate, the electrostatic repulsion, and the residence time of Au NPs at the interfacial region where the plasma interacted with the solution. It was revealed that the size and size distribution of the Au NPs could be tuned by adjusting the processing parameters of atmospheric microplasma-assisted electrochemical method.

## Methods

**Figure 1:**
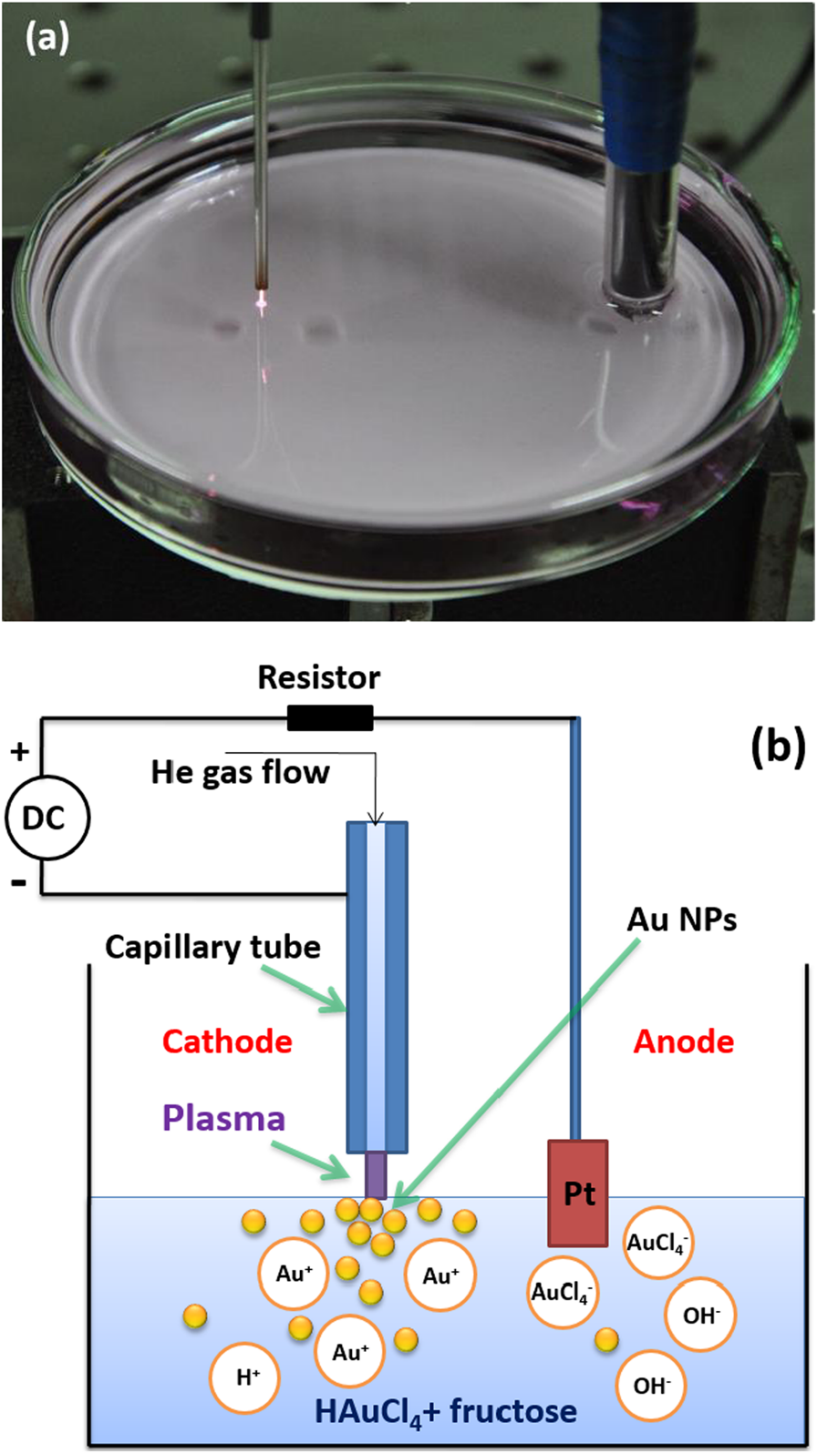
The experimental setup (a) together with its schematic (b) for atmospheric microplasma-assisted synthesis of Au nanoparticles.

The experimental setup together with its schematics for atmospheric microplasma-assisted synthesis of Au NPs are shown in Figure 1. A 10-ml solution of 0.2-mM HAuCl_4_ and 0.01-M fructose were placed in a petri dish, in which HAuCl_4_ would be deoxidized into Au particles, and fructose was a stabilizer that prevented uncontrolled particle growth and agglomeration. The stainless steel capillary tube (175-μm inside diameter; 5-cm length) and the metal foil of Pt (1 × 1 cm^2^; 0.001-in. thick) worked as cathode and anode, respectively. The cathode positioned 3 cm away from the anode with a gap of 2 mm between the tube end and the liquid surface. Pure helium (25 SCCM; 99.999% purity) was coupled to the capillary tube. To ignite the microplasma, a high voltage of 2 kV (DC power, purchased from Dongwen Corporation in Tianjin, China) was applied; following gas breakdown, the discharge current was kept constant for the duration (15 min) of the experiment. The liquid changes color (indicating colloidal metal nanoparticle growth) within a few minutes as the microplasma impinges directly onto the surface of the solution.

Particle growth was monitored by UV-Vis absorbance spectroscopy (AvaSpec-2048-2-USB2) in which the background spectrum from de-ionized water was used as a reference. Nanoparticles were drop-cast and dried in the room atmosphere on carbon-coated copper TEM grids, and the sizes and morphologies of as-grown NPs were characterized by TEM.

## Results and discussion

**Figure 2:**
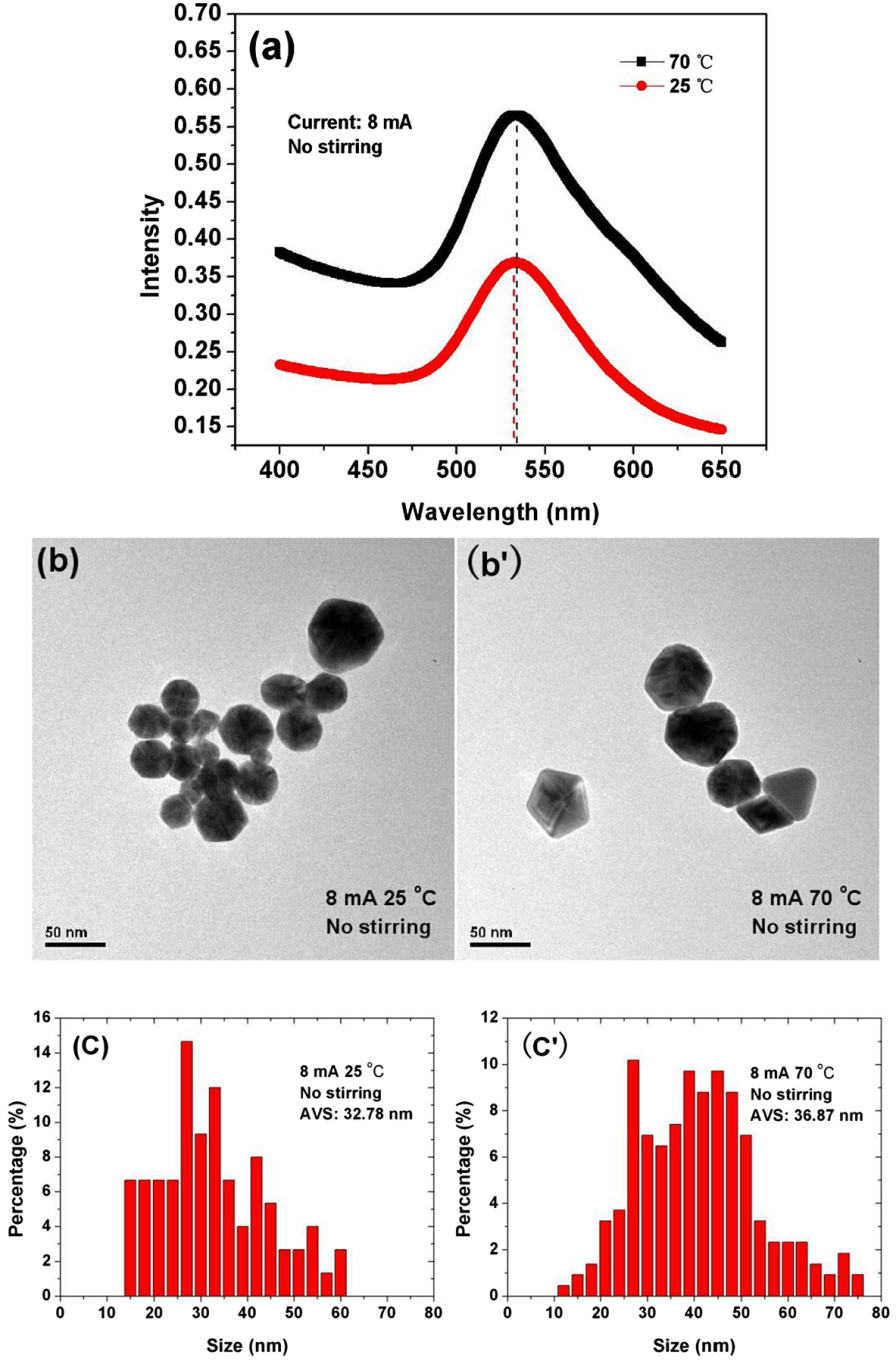
**UV–Vis absorbance spectroscopy (a), TEM images (b, b’), and PSD (c, c’) of Au NPs.** Synthesized at temperatures of 25°C and 70°C, using 8 mA of current with non-stirring condition.

The UV-Vis absorbance spectroscopy of Au particles at room temperature (25°C) and high temperature (70°C) are shown in Figure 2a. Both experiments without stirring were carried out at a current of 8 mA. The UV-Vis absorbance spectroscopies show that the most probable size of Au NPs produced at different temperature is nearly the same since both of the absorption peaks are around 530 nm. The absorbance curve of Au NPs produced at 70°C shows a little upward trend at the long wavelength, reveals that the higher temperature enhances the growth of the larger Au NPs; nevertheless, the influence of the temperature is not large enough so that it cannot make the peak of the absorbance curve red-shifted.TEM images and PSD of the Au NPs produced at temperature of 25°C and 70°C are shown in Figure 2b,b’ and c,c’, respectively. The average size (AVS) is 32.78 nm for the Au NPs produced at 25°C and 36.87 nm for the Au NPs produced at 70°C. Although the AVS of Au particles fabricated at 70°C is larger, the most probable sizes of Au NPs produced at both temperatures are nearly the same. The result agrees well with that given by UV-Vis absorbance (Figure 2a).

As for the preparation of Au NPs, there is the balance equation:

$$H\left[{\mathrm{AuCl}}_{4}\right]⇌{\mathrm{AuCl}}_{3}+HCl\text{;}$$

the Au NPs are produced from the reduction of Au^3+^ ions (from AuCl_3_) instead of [AuCl_4_]^-^ complex. The place where the plasma contacts a liquid is named as the interfacial region. As a rise of temperature, more HAuCl_4_ decompose into Au^3+^ ions, and the increase of the concentration of Au^3+^ ions in solution will enhance the production of Au NPs. With more Au NPs being produced at the interfacial region, large numbers of Au NPs with enormous size are produced due to the agglomeration of Au NPs at the interfacial region.

On the other hand, according to the Stokes-Einstein equation for the diffusion coefficient of the nanoparticles in solution [31]:

$$D_{iB}^{\infty }\approx \frac{kT}{6\pi \eta_{B}r_{i}}\text{,}$$

where *k* is the Boltzmann constant, *T* is the temperature of the liquid, *η*_*B*_ is the viscosity of the fluid, and *r*_*i*_ is the radius of the Au particles. By increasing the temperature, the diffusion coefficient increases; as a consequence, the residence time of Au NPs at the interfacial region gets down, which weakens the agglomeration of Au NPs at the interfacial region and boosts the production of the small Au NPs.

**Figure 3:**
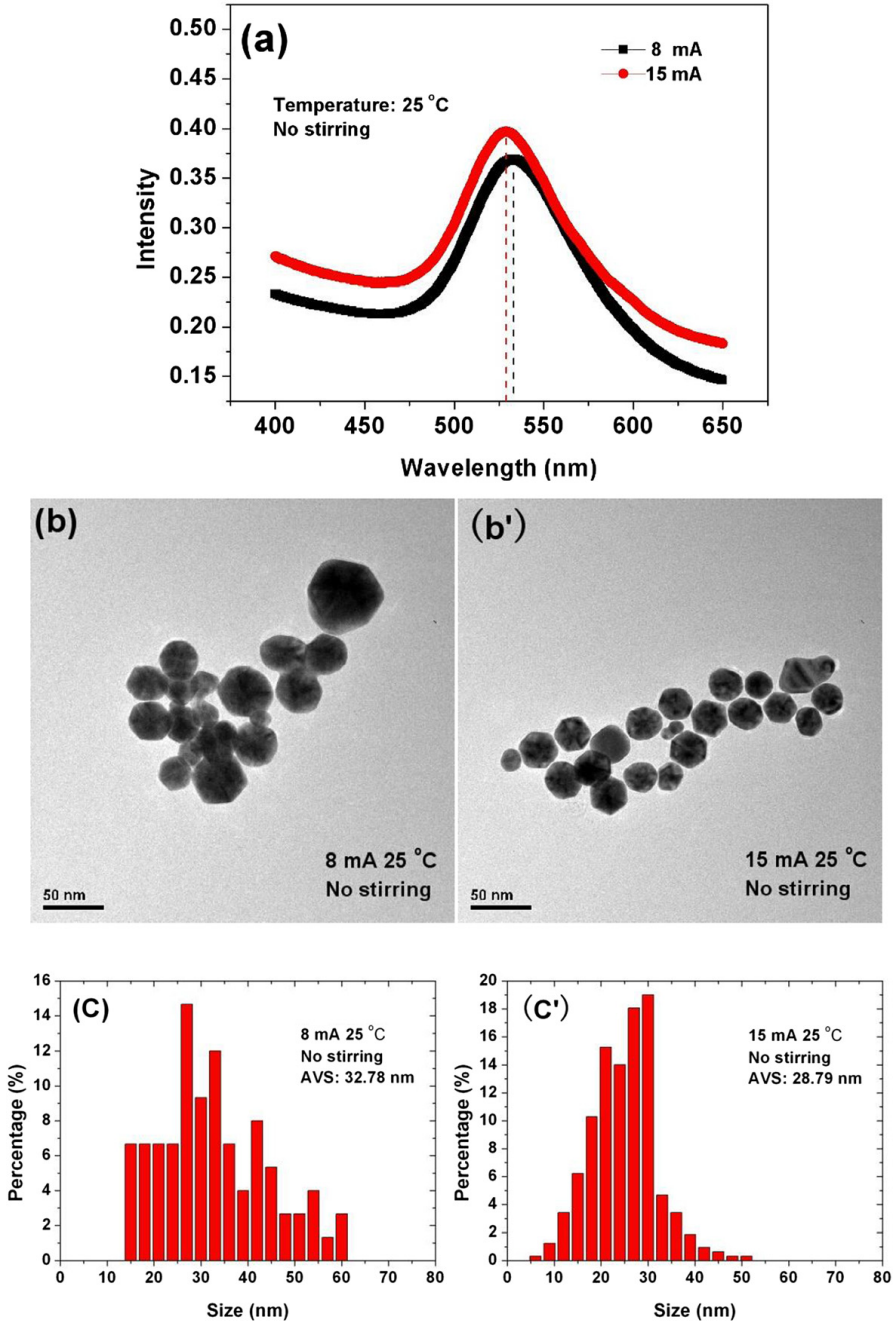
**UV–Vis absorbance spectroscopy (a), TEM images (b, b’), and PSD (c, c’) of Au NPs.** Synthesized at room temperature of 25°C, using (8, 15) mA of currents with non-stirring condition.

Obviously, by increasing the temperature, there are two opposite factors that affect the growth of Au NPs: one is due to the increase of the Au^3+^ ions concentration and another is due to the decrease of the residence time of Au NPs at the interfacial region. The former will do a favor to produce large Au NPs, whereas the latter will favor the small NPs production. The relative stronger effect of the increase of the Au^3+^ ions concentration ultimately results in more Au NPs with large size produced as shown in Figure 2b,b’ and c,c’, though it is not strong enough to change the most probable size of the Au NPs thus produced.Figure 3a shows the UV-Vis absorbance spectroscopy of Au colloids produced at the current of 8 mA and 15 mA by using the atmospheric microplasma electrochemistry. Both experiments were carried out at room temperature without any stirring. The blue shift of the UV-Vis absorption peak for the Au NPs produced at 15 mA indicates that the most probable size of nanoparticles fabricated at higher values of current is smaller.TEM images and PSD of the Au NPs produced at a current of 8 mA and 15 mA are given in Figure 3b,b’ and c,c’, respectively. The average size of the Au NPs produced at 8 mA is 32.78 nm, and that at 15 mA is 28.79 nm and it can be seen that that Au NPs produced at higher values of current have smaller average size. The results agree well with that given by UV-Vis absorbance spectroscopy. Both the results show that the Au NPs produced at the higher values of current have the smaller average size as compared to that produced at the lower values of current.

**Figure 4:**
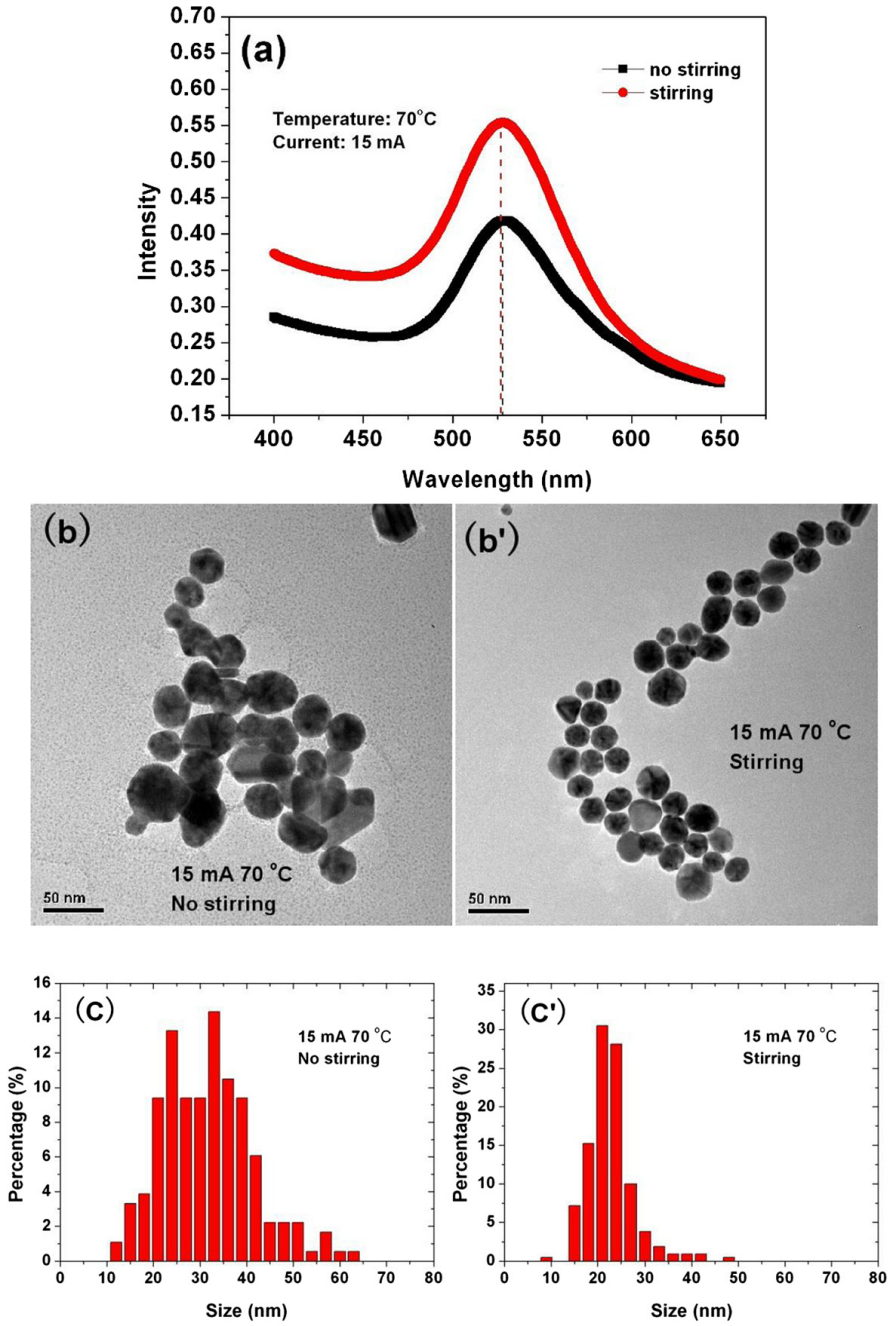
**UV–vis absorbance spectroscopy (a), TEM images (b, b’), and PSD (c, c’) of Au NPs.** Synthesized at a temperature of 70°C, using 15 mA of current with and without stirring condition.

As the current increases, more electrons are injected from plasma into the solution per unit time; as a result, more Au NPs are produced per unit time, i.e. the production rate of Au NPs increases. Since not all the electrons injected by the plasma are involved in Au^3+^ reduction [32], the remaining electrons are involved in the charge-transfer process such as charging the produced Au NPs at the interfacial region [30]. Obviously, the higher current leads to the higher production rate of Au NPs and as a result, the more and more Au NPs are being charged. The charged Au NPs repel each other, impede the agglomeration and the growth of the Au NPs, and lead to the production of the Au NPs with smaller size.Figure 4a presents the UV-Vis absorbance spectroscopies of the Au colloids produced at the condition with and without stirring. Both of experiments were carried out at the current of 15 mA and the temperature of 70°C. The stirring reduces the UV-Vis absorbance at the longer wavelength which specifies the decrease of Au NPs with large size; nevertheless, clear blue shift of the absorption peak is not identified. Figure 4b,b’,c,c’ shows TEM images and PSD of Au colloids produced at the condition with and without stirring, respectively. Both TEM images and PSD of Au NPs illustrate that the NPs synthesized under the condition with stirring are relatively small and uniform.

If producing Au NPs at the condition without stirring, the solution at the interfacial region turns from colorless to red rapidly at once as the plasma impinges on the solution, and then followed by the solution nearby the interfacial region turns red, finally the whole solution becomes red. The phenomenon reveals that the Au NPs produced at the interfacial region diffuse slowly from the interfacial region to the other place of the solution if no stirring is performed during the Au NPs production. However, while producing Au NPs at the condition with stirring, the whole solution turns from colorless to pale red at once as the plasma impinges on the solution and becomes red as the processing time is going on. This clearly illustrates that the Au NPs are thus produced and quickly diffused from the interfacial region to the other regions within the solution, i.e., the residence time of NPs at the interfacial region decreases due to stirring. Obviously, stirring decreases the residence time of Au NPs at the interfacial region and restrains the agglomeration and the growth of Au NPs at the interfacial region and is good for the production of Au NPs enjoying the small and uniform size.

## Conclusion

**Figure 5:**
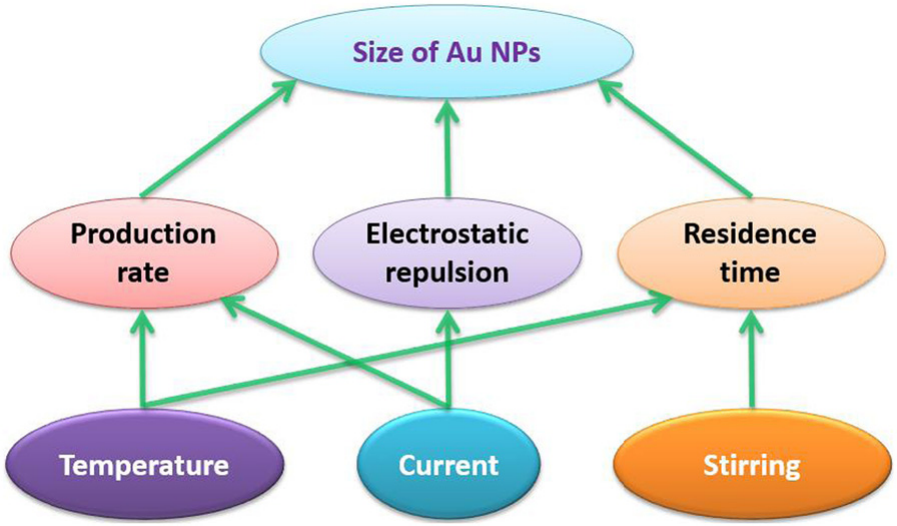
Schematic of deterministic processes for atmospheric microplasma-assisted synthesis of Au nanoparticles.

In this paper, the size of Au NPs synthesized by the atmospheric microplasma electrochemistry was studied as a function of solution temperature, current, and stirring, and the main results are summarized in Figure 5. As the temperature is raised, the increase in the growth rate of Au NPs caused by concentration of Au^3+^ ion plays a relatively stronger effect on the size and the size distribution of Au NPs compared to the reduction in the residence time of Au NPs at the interfacial region, which is caused by the increase in the diffusion coefficient. As a result of the increase of the current, more electrons are injected into the solution to reduce the Au^3+^ and charge the produced Au NPs, and more Au NPs with the relatively smaller size are produced under the effect of the electrostatic repulsion of the charged Au NPs. Stirring will decrease the residence time of Au NPs at the interfacial region and lead to the production of Au NPs with small and uniform size. The production rate, the electrostatic repulsion, and the residence time of Au NPs at interfacial region are expected to be the main factors that affect the size and size distribution of Au NPs. Further investigations are under way.

## Abbreviations

NPs: nanoparticles; TEM: transmission electron microscopy; PSD: particle size distribution; UV-Vis: ultraviolet-visible.

## Competing interests

The authors declare that they have no competing interests.

## Authors’ contributions

XH participated in the design of the study, performed the statistical analysis, and drafted the manuscript. YL participated in the design of the study and helped to perform the statistical analysis. XZ conceived of the study and participated in its design and coordination and helped to draft the manuscript. All authors read and approved the final manuscript.
